# NEDD4L Is Downregulated in Colorectal Cancer and Inhibits Canonical WNT Signaling

**DOI:** 10.1371/journal.pone.0081514

**Published:** 2013-11-28

**Authors:** Jarred P. Tanksley, Xi Chen, Robert J. Coffey

**Affiliations:** 1 Department of Cell and Developmental Biology, Vanderbilt University, Nashville, Tennessee, United States of America; 2 Department of Biostatistics, Vanderbilt University, Nashville, Tennessee, United States of America; 3 Department of Medicine, Vanderbilt University, Nashville, Tennessee, United States of America; 4 Department of Veterans Affairs Medical Center, Nashville, Tennessee, United States of America; University of Kentucky, United States of America

## Abstract

The NEDD4 family of E3 ubiquitin ligases includes nine members. Each is a modular protein, containing an N-terminal C2 domain for cell localization, two-to-four central WW domains for substrate recognition, and a C-terminal, catalytic HECT domain, which is responsible for catalyzing the ubiquitylation reaction. Members of this family are known to affect pathways central to the pathogenesis of colorectal cancer, including the WNT, TGFβ, EGFR, and p53 pathways. Recently, *NEDD4* mRNA was reported to be overexpressed in colorectal cancer, but tumor stage was not considered in the analysis. Expression of the other family members has not been studied in colorectal cancer. Herein, we determined the expression patterns of all nine NEDD4 family members in 256 patients who presented with disease ranging from premalignant adenoma to stage IV colorectal cancer. *NEDD4* mRNA was significantly increased in all stages of colorectal cancer. In contrast, *NEDD4L* mRNA, the closest homolog to *NEDD4*, was the most highly downregulated family member, and was significantly downregulated in all tumor stages. We also found NEDD4L protein was significantly decreased by western blotting in colorectal cancer samples compared to adjacent normal mucosa. In addition, NEDD4L, but not catalytically inactive NEDD4L, inhibited canonical WNT signaling at or below the level of β-catenin *in vitro*. These findings suggest that NEDD4L may play a tumor suppressive role in colorectal cancer, possibly through inhibition of canonical WNT signaling.

## Introduction

Colorectal cancer (CRC) is the third most common cancer in the United States by incidence, and second only to lung cancer in mortality, causing approximately 50,000 deaths each year [1]. The process of tumorigenesis in sporadic CRC typically begins with an inactivating mutation in the *Adenomatous Polyposis Coli (APC)* gene that results in aberrant activation of the canonical WNT signaling pathway [2]. In the remaining cases, there is often a mutation in *CTNNB1*, which encodes β-catenin [3]. The result of each of these mutations is the diminution or loss of a cell's ability to properly target cytoplasmic β-catenin to the multi-protein E3 ubiquitin ligase, beta-transducin repeat-containing protein (β-TRCP), which poly-ubiquitylates β-catenin, and targets it to the proteasome for degradation [4]. Instead, β-catenin accumulates in the nucleus, and partners with the TCF/LEF transcription factor to drive a transcriptional program that ultimately results in neoplasia. In this scenario, it is the cell's inability to control levels of β-catenin through ubiqitin-mediated degradation that promotes tumorigenesis. However, the process of ubiquitylation as a whole is thought to be upregulated in cancer, which has led to the use of proteasome inhibitors in CRC treatment [5].

Ubiquitylation is a sequential, three-step process involving an E1, E2, and E3 ubiquitin ligase that results in the covalent linkage of ubiquitin(s), a 76 amino acid protein, to a Lys residue(s) of a substrate or target protein. It is central to cellular homeostasis, primarily by targeting proteins to the proteasome or lysosome for degradation via the attachment of ubiquitin chains (poly-ubiquitylation). However, ubiquitylation has also been shown to play important roles in the trafficking, localization, and activity of proteins, especially when there is the addition of only one ubiquityl moiety, termed mono-ubiquitylation [6]. Target specificity is primarily provided by E3 ligases, which catalyze the final step of ubiquitin attachment. In the human genome, there are over 600 E3 ligases, versus approximately 30 E2s and two E1s. A given E3 can have many targets, and can activate or inhibit multiple cellular signaling pathways [7]. There are two major families of E3s: the Really Interesting New Gene (RING) family, comprising 95% of all E3s, and the Homologous to the E6-AP Carboxyl Terminus (HECT) family, comprising the remaining 5% [8].

The NEDD4 family of HECT-type E3s includes nine members [9]. Each member is a modular protein, with an N-terminal C2 domain, two-to-four central WW domains, and a C-terminal, catalytic HECT domain. The WW domains are responsible for target selection, and interact with a four amino acid motif (PY motif, PPXY) in the target protein, while the C2 domain aids in proper cellular localization. The HECT domain is responsible for recruiting the E2, which serves as a carrier for ubiquitin and directly conjugates ubiquitin to the target Lys residue, a process that requires an active-site Cys residue. The founding member of this family, NEDD4, has recently been shown to be upregulated in CRC, and to promote the proliferation of CRC cells *in vitro*, though the mechanism remains unclear [10]. Beyond that study, nothing is known about the expression patterns of the NEDD4 family in CRC. Of note, many of the NEDD4 family members have been found to target proteins that are integral players in pathways thought to be most important in the genesis and progression of CRC.

In this study, we sought to determine mRNA expression levels of each of the nine NEDD4 family members in tumors from a large cohort of individuals with colorectal neoplasia. We found that *NEDD4* is the most highly upregulated member of the family. Surprisingly, *NEDD4L*, which shares the greatest sequence homology with *NEDD4*, was the most highly downregulated member of the family in CRC. We confirmed the downregulation of NEDD4L at the protein level in human CRC samples. Recently, NEDD4L was shown to degrade DVL2, thus inhibiting WNT signaling, as determined by reduced TOPFlash activity [11]. We also found that NEDD4L inhibits TOPFlash activity at or below the level of β-catenin. Taken together, these findings suggest that NEDD4L has the potential to act as a tumor suppressor in CRC.

## Materials and Methods

### Ethics statement

All of the human samples used in our manuscript were collected and analyzed in accord with a protocol approved by the Institutional Review Boards at Vanderbilt University Medical Center, Nashville, TN, and Moffitt Cancer Center, Tampa, FL. The samples used in this publication have been described previously [12], [13]. The resulting data were analyzed anonymously.

### Human tissue sample microarray analysis

A total of 250 human CRC tissue samples were collected at VUMC (N = 55) and MCC (N = 195). Of these CRC samples, 33 were Stage I (13.2%), 76 Stage II (30.4%), 82 Stage II (32.8%), and 59 Stage IV (23.6%). Adjacent normal tissue was collected at MCC from ten of these patients. Of these, two were collected from Stage I, five from Stage II, and three from Stage III patients. An additional six adenomas were collected at MCC. The samples used in this publication have been described previously [12], [13]. RNA from these tissues was obtained and hybridized to the Affymetrix Human Genome U133 Plus 2.0 GeneChip Expression Array. The following probes were analyzed: *NEDD4* (213012_at), *NEDD4L* (237498_at, 241396_at, 212445_s_at, 212448_at), *WWP1* (212637_s_at, 212638_s_at), *WWP2* (1552737_s_at, 1554580_a_at, 204022_at, 210200_at), *ITCH* (209743_s_at, 209744_x_at, 217094_s_at, 235057_at, 239101_at), *SMURF1* (1559426_at, 212666_at, 212668_at, 215458_s_at, 232665_x_at), *SMURF2* (205596_s_at, 227489_at, 232020_at), *NEDL1* (210331_at, 215584_at), and *NEDL2* (232080_at, 243080_at). Of the four probes specific to *NEDD4L*, we focused our attention on 212445_s_at as it encodes a portion of the translated protein. Three of the four NEDD4L probes were significantly downregulated in a similar manner, while one (237498_at) remained unchanged with progression.

Patients in the VUMC group had a median follow-up of 50.2 months, with a minimum follow-up of 0.4 months and a maximum of 111.3 months. Those in the MCC group had a median follow-up of 44.7 months, with a minimum of 0.92 and a maximum of 142.8 months. All of these samples were collected and analyzed in accord with a protocol approved by the Institutional Review Boards at both VUMC and MCC.

### Western blot analysis

CRC tissue and adjacent normal colonic mucosa samples were collected from 20 individuals at surgical resection at VUMC and snap frozen in liquid nitrogen. Samples were then lysed in 8 M urea, sonicated, and centrifuged at 14,000 rpm. The supernatant was removed, and the protein quantified by measuring absorbance at 280 nm. The samples were then run on a 7.5% polyacrylamide gel and transferred to nitrocellulose membranes. The membranes were blotted with anti-NEDD4L (1∶2000) (Bethyl Laboratories, Montgomery, TX) and anti-α-tubulin (1∶2000) (TUBA) (CalBiochem, San Diego, CA) antibodies, and detected using appropriate HRP-conjugated secondary antibodies and the chemiluminescence method. The intensities of the blots were then quantified using the ImageJ software, and analyzed.

### TOPFlash assay

Transfections were performed using Metafectene (Biontex, San Diego, CA) according to the manufacturer's instructions. HEK293 cells (2×10^5^) were plated in a 12-well dish, allowed to attach, and transfected the following day with 0.1 µg of each plasmid. The following plasmids were used: TOPFlash reporter plasmid, FOPFlash reporter plasmid, pcDNA3.1, NEDD4L (KIAA0439) (Addgene plasmid 27000), and NEDD4L(C>A) (Addgene plasmid 27001) [14]. Constructs containing wild-type (wt) β-catenin and mutant β-catenin (ΔN89) were a gift from Ethan Lee at Vanderbilt University. The day after transfection, the medium was removed, and serum-free medium containing 20 ng/ml WNT3A and 100 ng/ml R-spondin (Vanderbilt Antibody and Protein Shared Resource) was added. The following day, cells were lysed and processed in accord with the luciferase assay kit (Promega, Fitchburg, WI). TOPFlash activity was normalized to FOPFlash activity, and fold-activation was determined by normalizing to TOPFlash and FOPFlash activity in unstimulated cells. All experiments were performed in triplicate at least three times.

### NEDD4L knock-down

Lentiviral plasmids containing shRNA (Open Biosystems, Huntsville, AL) against human *NEDD4L* were transfected into HEK293 cells using Metafectene. The following day, fresh medium was added. The next day the medium was filtered (0.45 µm filters). To infect DLD-1 and RKO cells, 2.0×10^5^ cells were plated in each well of a 6-well plate, and infected with the lentivirus. After two days, GFP expression was observed, and infected cells were selected with puromycin (5 µg/ml) and sorted for GFP expression. Efficiency of knock-down was determined by NEDD4L western blotting.

### Statistical analysis

To compare the expression level of each probe between normal, adenoma, and the cancer tissue samples, the Wilcoxon rank sum test was used [12], [13]. To determine relative protein levels, NEDD4L protein levels were normalized to TUBA levels, log-transformed, and compared using a paired t-test. Kaplan-Meier survival analysis was performed on the microarray dataset in order to determine disease-specific survival. Disease-specific survival was defined as a documented cancer-related death [12]. Significance levels in the TOPFlash reporter assay experiment were determined using the Student's t-test.

## Results

### Expression levels of NEDD4 family members in CRC

**Figure 1:**
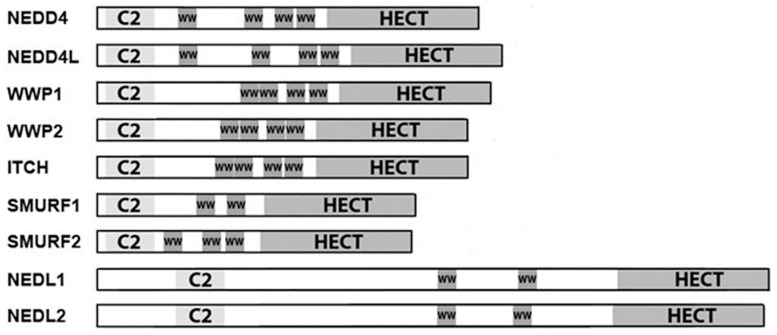
The NEDD4 family of E3 ubiquitin ligases. The nine members of the NEDD4 family are modular proteins. At their N-terminus, each has a C2 domain, which plays a role in proper cellular localization. The central portion of each protein contains two-to-four WW domains, which are responsible for target recognition. WW domains are 20–23 amino acid, Trp-based motifs that recognize PY motifs (PPXY, LPXY), as well as some phospho-Ser- or phospho-Thr-based motifs. At the C-terminus is the HECT domain, which directly conjugates an ubiquityl moiety to the target protein. The family members are grouped together by name and homology. ITCH is more closely homologous to WWP1 and 2 than to other family members.

The nine members of the NEDD4 family of E3 ubiquitin ligases are modular proteins (Figure 1). As a whole, the NEDD4 family is known to be involved in the regulation of a number of proteins and pathways that are central to the development of CRC. Table 1 is a compilation of known substrates, signaling pathways affected, and expression levels in various cancers of all the NEDD4 family members. There is high sequence homology amongst the family members, which explains the shared targets of many of the family members. However, there are also examples of different family members having opposing effects on the same signaling pathway. For example, NEDD4 causes the downregulation of SMAD1 and CBL, which inhibits bone morphogenetic protein (BMP) signaling and activates epidermal growth factor receptor (EGFR) signaling, respectively [15], [16]. On the other hand, NEDD4L impacts transforming growth factor-β (TGFβ) signaling, but not BMP signaling, through the degradation of SMAD 2 and 3 and the type I TGFβ receptor, and abrogates EGFR signaling through the ubiquitin-mediated degradation of activated CDC42 kinase 1 (ACK1) [14], [17], [18]. Additionally, NEDD4 has been shown to downregulate the tumor suppressor PTEN [19]. However, NEDD4 was shown to promote the proliferation of CRC cells *in vitro*, independent of an effect on PTEN levels [10].

**Table 1:** The known targets of the NEDD4 family, the signaling pathways affected, and their expression levels in human cancers.

E3	Known targets	Signaling pathways affected	Expression in cancer
NEDD4	CBL, PTEN, SMAD1, PTC, IGF1R, VEGFR2, FGFR1, Notch, ENaC, RNAPII, CNrasGEF, AR, Sprouty2, Beclin1, Bcl10, p63, p73	EGFR↑, PI3K/AKT↑, BMP↓, Hh↑, Notch↓	Colon↑[10], Gastric↑[28], Non-small cell Lung↑[29]
NEDD4L	ACK1, SMAD 2 & 3, TGFBR1, DVL2, ENaC, Kv1.3, SGLT1, NaV1.5, ATA2, Fe65, DAT, Occludin, TrkA, hERG1, p130	EGFR↓, TGFβ↓, WNT↓	Gastric↓[23], Prostate↑[24]/↓[25], Gallbladder↑[26], Glioma↓[27]
WWP1	CDKN1B, SMAD4, TGFBRI, HER4, p27, Ezrin, KLF5, JunB, RNF11	TGFβ↓, p27↓	Breast↑[30], Prostate↑[31]
WWP2	Oct4, PTEN, SMAD 2, 3 & 7, ENaC, EGR2, GluR2, Gsc	PI3K/AKT↑, TGFβ↓/↑	
ITCH	CBL, HER4, DVL2, p63, p73, CXCR4, Bcl10, HER4	EGFR↑, HER4↓, WNT↓, p53↓	
SMURF1	SMAD 1, 4, 5 & 8, TGFBR1, MDM2, STAT1, RhoA, ING2, JunB, Talin	BMP↓, TGFβ↓/↑, p53↓	Pancreas↑[32]
SMURF2	CBL, GSK3B, SMAD 1, 2, 3 & 7, Prickle, RNF11, SMURF1, Id1, KLF5	EGFR↑, TGFβ↓, BMP↓	Renal cell↑[33], Esophageal↑[34]
NEDL1	HER4, DVL2, p53	HER4↓, WNT↓, p53↑	Neuroblastoma↓[35]
NEDL2	p73	p53↑	

In the “Signaling pathways affected” and “Expression in cancer” columns, ↓ indicates inhibition and downregulation, respectively, while ↑ indicates activation and upregulation. Patched (PTC), epithelial sodium channel (ENaC), cyclic nucleotide ras GEF (CNrasGEF), androgen receptor (AR), B-cell CLL/lymphoma 10 (Bcl10), potassium voltage-gated channel, shaker-related subfamily, member 3 (Kv1.3), sodium/glucose cotransporter 1 (SGLT1), sodium channel, voltage-gated, type V (NaV1.5), amyloid beta A4 precursor protein-binding family B member 1 (Fe65), dopamine transporter (DAT), inhibitor of growth 2 (ING2).

**Figure 2:**
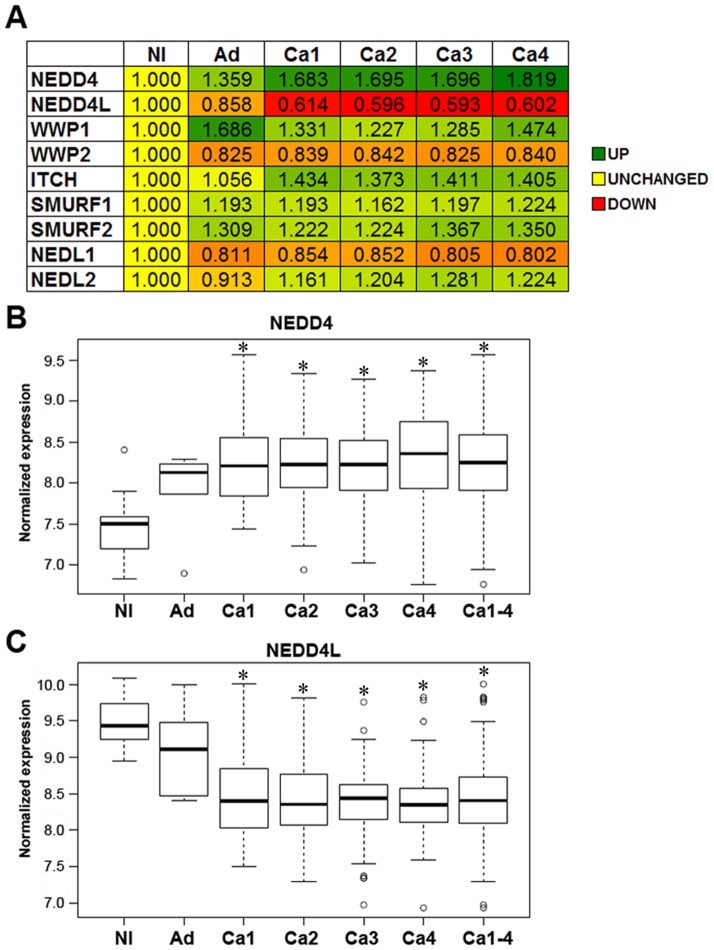
Expression levels of the NEDD4 family in CRC. (A) The expression of all nine NEDD4 family members was examined in CRC by microarray profiling. Shown here is the average fold-change of all probes for each individual gene in Nl (N = 10), Ad (N = 6), Ca1 (N = 33), Ca2 (N = 76), Ca3 (N = 82), and Ca4 (N = 59). (B) *NEDD4* (213012_at) is the most highly upregulated member of the NEDD4 family in CRC. (C) *NEDD4L* (212445_s_at) is the most highly downregulated member of the NEDD4 family in CRC. Nl =  normal, Ad =  adenoma, and Ca1-4 = CRC, stage I-IV. *p<0.05.

In order to determine a potential role for the NEDD4 family in the initiation or progression of CRC, we subjected 250 CRC patient tumor samples of differing stages, six adenoma samples, and 10 adjacent normal samples to microarray analysis, noting how the expression of each family member changed with progression (Figure 2A). Interestingly, the closely homologous pair of *NEDD4* and *NEDD4L* were oppositely regulated (Figure 2B, C). *NEDD4* (213012_at) trended upward at the adenoma stage, but was significantly elevated in Stage 1 CRC and remained significantly elevated during tumor progression. Conversely, *NEDD4L* (212445_s_at) expression decreased, trending downward in adenomas, and was significantly decreased in all stages of CRC (Figure 2C).

### NEDD4L protein is decreased in CRC

**Figure 3:**
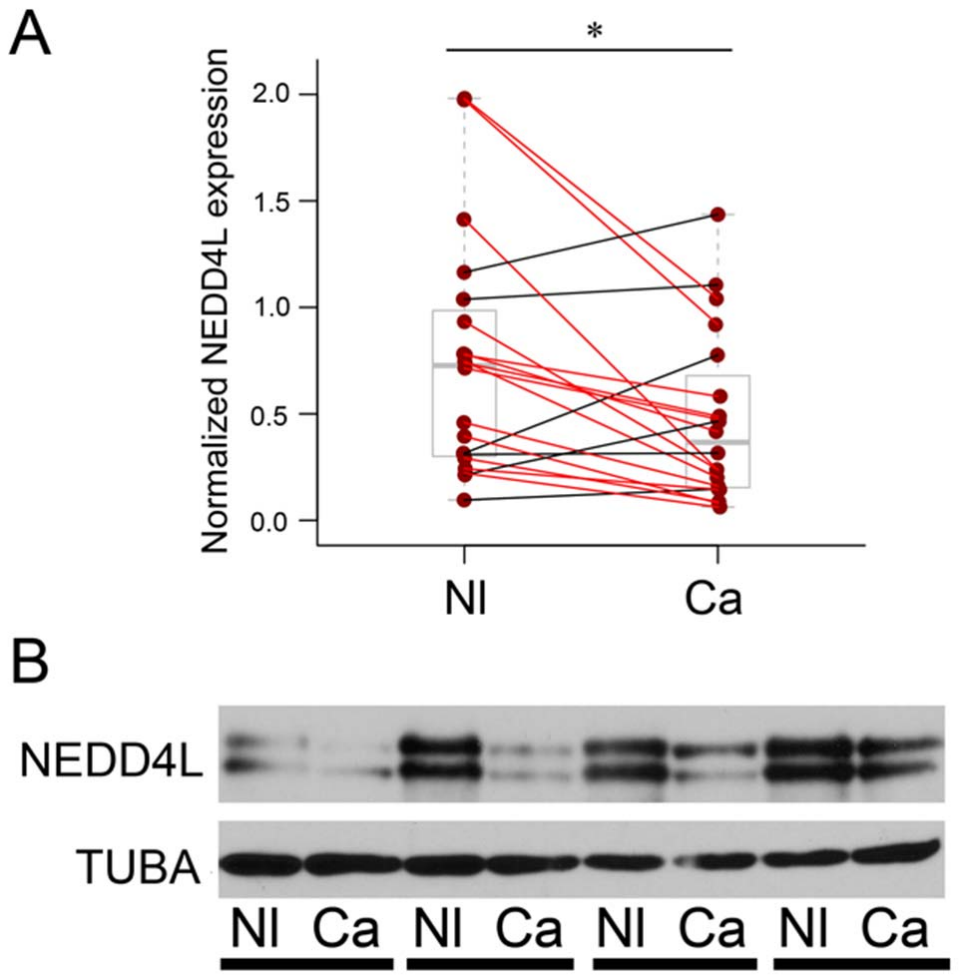
NEDD4L protein levels are down in CRC. (A) NEDD4L levels were determined by western blotting of CRC (Ca) and adjacent normal (Nl) mucosa from twenty individuals. Levels were normalized to TUBA and Nl was compared to Ca. NEDD4L was significantly downregulated (∼42%) in CRC (*p<0.05). Data is represented in box and whisker plot format. The lines connecting data points show the relative NEDD4L levels in a given Nl-Ca matched pair. Red denotes decreased NEDD4L levels in Ca, while black denotes an increase. (B) Lysates generated from Nl-Ca matched pairs were blotted for NEDD4L and TUBA. Shown here are four representative pairs.

As transcript levels of a particular gene do not always correlate with protein levels, we next determined whether the decrease in *NEDD4L* mRNA levels resulted in a decrease in NEDD4L protein. To do this, we performed western blot analysis on human CRC and adjacent normal tissue samples from 20 patients using tissue collected at Vanderbilt University Medical Center (Figure 3A, B). We observed a significant decrease in NEDD4L protein levels (∼42%) in human CRC compared to normal tissue, a finding consistent with the microarray analysis.

**Figure 4:**
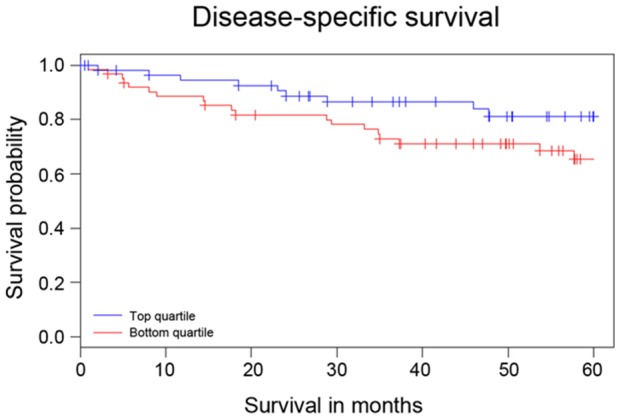
Patients with highest NEDD4L expression show a trend towards longer disease-specific survival compared to those with the lowest. Disease-specific survival in CRC patients with the highest and lowest NEDD4L expression was compared using Kaplan-Meier analysis. Those patients with tumor samples in the highest quartile of NEDD4L expression showed a trend towards longer disease-specific survival over a 60-month period. p = 0.079.

We next sought to determine whether NEDD4L expression levels correlate with disease-specific survival in our cohort of patients. We compared the survival of patients in the highest quartile of NEDD4L expression with that of those in the lowest quartile (Figure 4). We found that those patients with the highest NEDD4L expression exhibited a longer period of disease-specific survival than those patients with the lowest expression levels. This difference, however, did not reach statistical significance (p = 0.079).

### NEDD4L suppresses canonical WNT signaling

**Figure 5:**
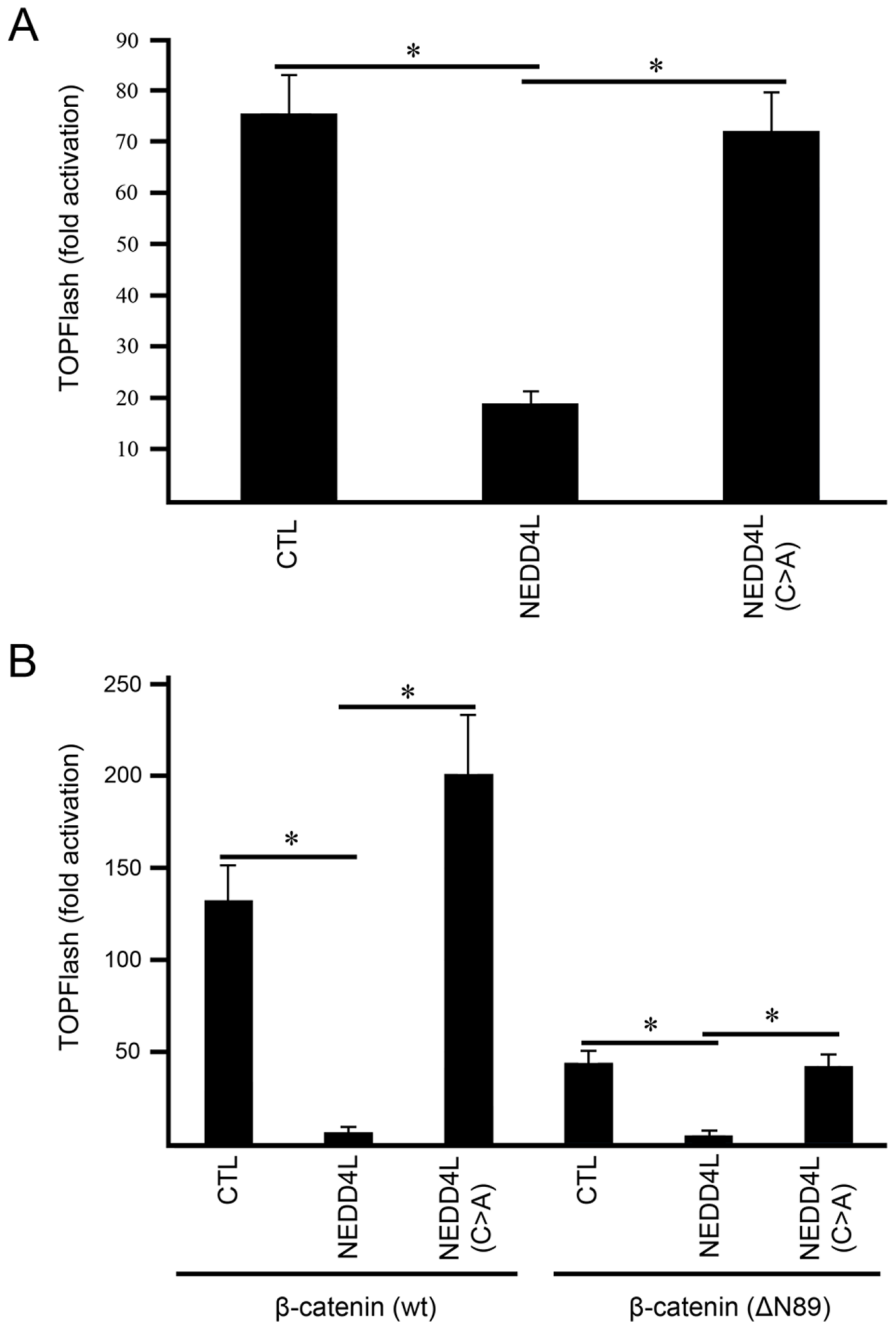
NEDD4L inhibits canonical WNT signaling at or below the level of β-catenin activation. (A) NEDD4L inhibits TOPFlash activity in HEK293 cells compared to empty vector (CTL) or catalytically inactive NEDD4L [NEDD4L (C>A)]. WNT3A (20 ng/ml) and R-spondin (100 ng/ml) were used to activate canonical WNT signaling. (B) When two different β-catenin constructs, β-catenin (wt) and β-catenin (ΔN89), are used to activate canonical WNT signaling a significant reduction in TOPFlash activity was observed in the presence of NEDD4L overexpression, as compared to empty vector or NEDD4L (C>A). All results are normalized to FOPFlash activity. *p<0.05.

The significant decrease in NEDD4L expression in CRC suggests the possibility that NEDD4L plays a tumor-suppressive role in CRC. Given the centrality of canonical WNT signaling to the initiation and progression of CRC, we investigated whether NEDD4L can suppress canonical WNT signaling by performing the TOPFlash reporter assay, which measures canonical WNT activity by fusing TCF/LEF response elements to a firefly luciferase reporter. NEDD4L inhibited TOPFlash activity when canonical WNT signaling was activated by the addition of WNT3A and the coactivator, R-spondin (Figure 5A). Inhibition appears to be dependent upon the ability of NEDD4L to ubiquitylate target proteins, as the catalytically inactive form of NEDD4L, in which the active-site Cys was mutated to an Ala [NEDD4L (C>A)] showed no inhibition of TOPFlash activity (Figure 5A) [20]. These results support the recent finding that NEDD4L inhibits WNT signaling by ubiquitylating and targeting DVL2 to the proteasome for degradation [11]. However, we also found that NEDD4L inhibited canonical WNT signaling when wt or mutant (ΔN89) β-catenin was used as an activator (Figure 5B), suggesting NEDD4L suppresses WNT signaling downstream of DVL in the presence of mutant APC or CTNNB1.

To extend this work, we examined the growth effects of knocking down endogenous NEDD4L in two human CRC cell lines, DLD-1 and RKO. DLD-1 cells contain a truncating mutation in *APC*, while RKO cells have neither a mutation in *APC* nor *CTNNB1*, but do have an inactivating mutation in *NKD1*, an inducible negative regulator of WNT signaling [21]. Knock-down was verified by western blotting (∼80%) (data not shown). We measured growth on plastic, and in soft agar and collagen. We observed no significant differences in growth in cells with NEDD4L knock-down compared to scrambled controls (data not shown). Additionally, NEDD4L knock-down had no effect on tumor size in nude mouse xenografts (data not shown).

## Discussion

The present study was performed to begin to understand if there is a role for members of the NEDD4 family of E3 ubiquitin ligases in CRC. Nine members comprise this family, and each has been shown to impact at least one of the cellular pathways central to the initiation and progression of CRC. Several members have been shown to be dysregulated in a number of different cancers. However, a systematic evaluation of the expression levels of all family members in human CRC has not previously been performed. Notably, *NEDD4* mRNA expression was recently shown to be upregulated in CRC, but this analysis did not consider the stage of CRC, or include adenomas [10]. We felt that the first step in beginning to understand this family of E3s in CRC was to examine the expression levels of each family member in CRCs from a large cohort of patients. We chose to perform our analysis across all stages, including adenomas, as there are commonly alterations in similar pathways at similar points during CRC progression [2]. Some of these pathways have different functions at different stages. For instance, the TGFΒ pathway is a tumor suppressor early in CRC, but promotes progression and metastasis later [22].

Herein, we evaluated how the expression of each NEDD4 family member changes with progression from adenoma to Stage IV CRC. The most highly upregulated family member was *NEDD4*, in accord with the aforementioned findings [10]. We go on to show that the upregulation occurs early in CRC (stage I), and upregulation is maintained during progression. The most highly downregulated family member in our data set was *NEDD4L*. We found this surprising given that NEDD4L shares the highest sequence homology with NEDD4, and has been shown to have a significant overlap with NEDD4 in target selection [7]. Recently, NEDD4L protein levels were shown to be downregulated in gastric cancer, and those patients with the lowest expression by immunohistochemical analysis had a worse prognosis [23]. NEDD4L has also been found to be both upregulated and downregulated in prostate cancer, increased in invasive gallbladder cancer cells, and downregulated in more aggressive malignant gliomas [24]–[27]. These disparate findings are not unexpected given the number of targets and cell signaling pathways upon which NEDD4L has been found to act.

A recent report showed that NEDD4L inhibits both the canonical and non-canonical WNT signaling pathways by ubiquitylating DVL2, and targeting it to the proteasome for destruction [11]. Here, we found that NEDD4L can also inhibit canonical WNT signaling at the level of, or downstream from, β-catenin. This is in contrast to the aforementioned paper, in which the investigators showed that NEDD4L could not inhibit TOPFlash when signaling was activated by a β-catenin mutant (S37A). In their study, isoform 2 of NEDD4L (NM_001144964.1) was used, whereas we used a different NEDD4L isoform (KIAA00439). These two cDNAs differ at their N-terminus; the construct used in our study has an additional 141 amino acid residues at the immediate N-terminus, which modifies the C2 domain, and 20 fewer residues in a segment preceding the second WW domain. This might contribute to differential localization or substrate targeting. Additionally, different β-catenin constructs were tested; we used wt and mutant β-catenin (ΔN89), and, in the earlier report, β-catenin (S37A) was used.

We found that knocking down the expression of endogenous *NEDD4L* in two CRC lines had no effect on growth. This could be due to the fact that these cell lines have already evolved a mechanism to overcome the growth-inhibitory effects of NEDD4L, or that the cells have already achieved an endogenous reduction in NEDD4L expression to a level below that necessary for growth inhibition. Future studies will address this by overexpressing wt NEDD4L in a battery of CRC lines. Ultimately, it may be necessary to create a mouse model in which NEDD4L is knocked-out early in the process of tumorigenesis.

In conclusion, we have examined mRNA expression of the NEDD4 family of E3 ubiquitin ligases in a large cohort of individuals with colorectal neoplasia. The most highly upregulated member is *NEDD4*, which has been previously shown to enhance proliferation of human CRC cells *in vitro* [10]. The most highly downregulated member is *NEDD4L*. In agreement with previous findings, NEDD4L overexpression in HEK293 cells inhibited canonical WNT signaling [11]. Here, we also show that NEDD4L inhibits WNT signaling at or downstream of β-catenin, which is important in the context of the most common activating mutation in canonical WNT signaling that are found in CRC. Therefore, we propose that NEDD4L may act as a tumor suppressor in CRC by inhibiting canonical WNT signaling.
